# Warm Edge Kelp Populations Show Elevated Volatility to Marine Heatwaves

**DOI:** 10.1111/ele.70307

**Published:** 2026-01-23

**Authors:** Jiaxin Shi, Scott Bennett, Jules B. Kajtar, Thomas Wernberg, Neville S. Barrett, Graham J. Edgar, Neil J. Holbrook

**Affiliations:** ^1^ Institute for Marine and Antarctic Studies University of Tasmania Hobart Tasmania Australia; ^2^ Australian Research Council Centre of Excellence for Climate Extremes University of Tasmania Hobart Tasmania Australia; ^3^ National Oceanography Centre Southampton UK; ^4^ UWA Oceans Institute and School of Biological Sciences University of Western Australia Crawley Western Australia Australia; ^5^ Institute of Marine Research Flødevigen Research Station His Norway; ^6^ Australian Research Council Centre of Excellence for the Weather of the 21st Century University of Tasmania Hobart TAS Australia

**Keywords:** *Ecklonia radiata*, local adaptation, macroecology, niche conservatism, population dynamics, seaweeds, thermal performance

## Abstract

Reliable predictions of species responses to intensifying temperature extremes are crucial for managing climate change impacts. However, limited data of species' responses to heat stress across their distribution restricts prediction accuracy. Here we analyse three‐decades of kelp abundance observations in Australia, including cool to warm‐edge populations, relative to marine heatwaves (MHWs). As MHWs intensified, changes in kelp abundances shifted from positive to negative. Warm‐edge populations displayed steeper declines in abundance change than central and cool‐edge populations under comparable MHWs. Our results support a hybrid thermal performance model, whereby thermal limits differ between populations, but performance volatility increases toward species' warm‐edge, heightening vulnerability of warm‐edge populations. Importantly, realised impacts of MHWs were evident at smaller thermal anomalies than predicted by experiments and distribution models, highlighting the importance of calibrating theoretical approaches with realised ecological change. By integrating a multi‐faceted approach, our study is generalisable for improving predictions of species' population vulnerability.

## Graphical abstract

Limited data on how heat stress affects species across their distribution restricts prediction accuracy. Our long‐term reef observational data supports a hybrid thermal performance model, whereby thermal limits differ between populations, but performance volatility increases toward species' warm‐edge, heightening vulnerability of warm‐edge populations. Importantly, realised impacts of marine heatwaves were evident at smaller thermal anomalies than predicted by experiments and distribution models, highlighting the importance of calibrating theoretical approaches with realised ecological change.

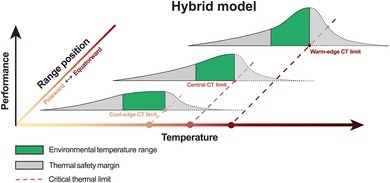

## 1 Introduction

Marine heatwaves (MHWs) can have devastating consequences for marine ecosystems and species, causing widespread socioeconomic and environmental impacts (Smith et al. 2024; Smith et al. 2021; Smale et al. 2019). Impacts include foundation habitat loss (Arias‐Ortiz et al. 2018; Hughes et al. 2018; Wernberg, Bennett, et al. 2016), impacts to aquaculture (Shi et al. 2024; Clement et al. 2016), and reduced catch rates in fishery species (Kajtar et al. 2024; Kendrick et al. 2019). In the context of climate change, MHWs have been increasing in duration, intensity and frequency (Oliver et al. 2018), a pattern that is projected to continue into the future (Holbrook et al. 2020; Oliver et al. 2019; Frölicher et al. 2018). It is not therefore surprising that these factors are severely stress‐testing species resilience (Pigot et al. 2023; Oliver et al. 2019).

Given the profound role of MHWs in shaping marine species and ecosystems, predicting species' responses to intensifying warming is crucial for management and conservation planning and has become a rapidly growing field of research (Assis et al. 2022; Harvey et al. 2022; Martínez et al. 2015; Sunday et al. 2012). Here, we consider the importance of short‐term intensifying MHWs on impacts to kelp across different populations of the same species. Various empirical approaches—including species distribution models, thermal performance experiments and field observations—have been employed to evaluate species' thermal responses. Each of these methods offers distinct insights, but they also come with specific assumptions that can influence their predictions (Table 1). Reconciling empirical evidence with mechanistic understanding of their differences and calibrating predictions against realised ecological change is essential for species' vulnerability and risk assessment (Pigot et al. 2023; Boyce et al. 2022). The key sources of uncertainty when predicting impacts of MHWs are the degree of variation in thermal performance across a species range (Bennett et al. 2019; Munday et al. 2013; Gaston et al. 2008; Vernberg 1962) and how species' thermal performance principles relate to MHW impacts in natural populations.

**TABLE 1:** Overview of the pros and cons for three empirical approaches commonly used to identify species' thermal performance. Each approach differs in its ability to capture mechanistic processes, ecological realism and predictive transferability.

Approaches	Pros	Cons
Laboratory experiments	Allow for mechanistic understanding and direct testing of causal links between climate drivers and ecological responses of species or populations (Vranken et al. 2021; Martínez et al. 2015; Peck et al. 2009)–particularly for multifactorial experiments (Boyd et al. 2018; Gunderson et al. 2016). Thermal performance curves can be clearly established (Wernberg, Bennett, et al. 2016). Possible to differentiate thermal performance between populations (Wernberg, de Bettignies, et al. 2016; Mabin et al. 2013; Staehr and Wernberg 2009). Lab‐based operations are easily replicated for small sized species, allowing for robust statistical analyses (Bates and Morley 2020). Feasible to test interactions with other stressors (Wernberg and Straub 2024; Bass et al. 2021).	The simplified conditions may yield relative thermal performance estimates that overestimate species' thresholds or underestimate climate change impacts compared with realised observations in the wild (Bates and Morley 2020; Morley et al. 2012; Wolkovich et al. 2012). Laboratory conditions limit ecological realism and neglect environmental interactions, variability and timescales (Dudney et al. 2025; Khelifa et al. 2019; Boyd et al. 2018; Wernberg et al. 2012). Outcomes can be highly sensitive to methodological decisions (e.g., acclimation period, heating rates), leading to variable results across studies (Smith et al. 2025; Bates and Morley 2020; Comte and Olden 2017). Short‐term experiments may not capture species' long‐term responses, including acclimation or adaptation consequences (Hemraj and Russell 2024; Beers and Sidell 2011; Peck et al. 2009).
Geographical distribution	Global databases (such as Ocean Biodiversity Information System or Global Biodiversity Information Facility) provide long‐term wide‐range occurrence data, including historical data, allowing for temporal and historical analyses at broad scales (Smale et al. 2019). Long‐term mean environmental data provide robust baselines for broad climatic envelopes and identifying large‐scale biogeographic patterns (Klaassen et al. 2025; Stewart et al. 2021). Real‐world ecological limits can be captured (Stuart‐Smith et al. 2015).	Limited insight into species responses to extreme temperatures and acute variability, especially population‐level difference (Perez‐Navarro et al. 2022; Bennett et al. 2019; Bateman et al. 2012; Marba and Duarte 2010). Local microclimates and short‐term tolerance beyond calculated range limits may lead to underestimated resilience or failure to capture the true thresholds (Carnicer et al. 2021; Peck et al. 2009). Range‐based observational approaches may produce predictions that diverge from observed outcomes, as they typically ignore acclimation capacity and dispersal processes (Starko et al. 2024; Woods et al. 2015; Potter et al. 2013). Biases of thermal preference estimation due to frequent collection of species occurrence records from easily accessible locations (Peck et al. 2009). Lack of knowledge of mechanism and cause‐effect underlying performances and distribution of species and ecosystems (Siegel and Dee 2025; Martínez et al. 2015; Peck et al. 2009).
In situ observation	Capture realised impacts in natural systems including complex intraspecific and interspecific ecological interactions (Bass et al. 2021; Wernberg, Bennett, et al. 2016). Allow for long‐term observation capturing trends, rare extreme events and lagged responses (Dudney et al. 2025; Reuman et al. 2025). In situ surveys often conducted at a fine spatial scale, provide high‐resolution data that reduce observational gaps within the study area (Hughes et al. 2018; Hughes et al. 2017). Population dynamics and climate context dependence can be revealed and compared (Lenoir 2020; Bennett, Wernberg, Arackal Joy, et al. 2015).	Population or ecosystem responses in the field (e.g., abundance change) typically slower than experimental controlled growth and production (Smale et al. 2019; Luo et al. 2011). The multifactorial nature with complex ecological interactions means isolating the effects of climate and removing confounding factors can be challenging (Dudney et al. 2025; Siegel and Dee 2025; Catford et al. 2022; Bennett et al. 2021; Ling 2008). Causal attribution is challenging when limited data and measurement bias undermine statistical power (Dudney et al. 2025; Siegel and Dee 2025). Retrospective in scope, constrained by the spatially and temporally limited occurrence of ecologically damaging warming events (e.g., MHWs), which restricts the predictive power.

**FIGURE 1:**
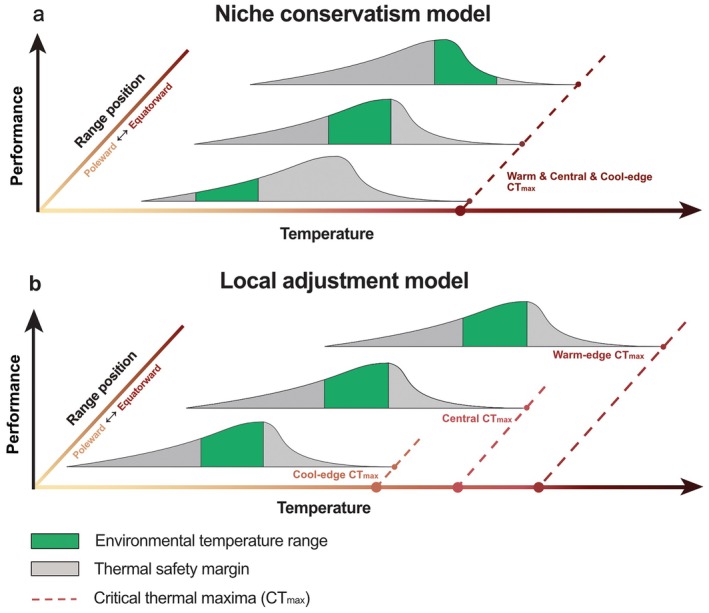
Heuristic models highlighting two potential relationships between temperature and physiological performance across different geographical range positions within a given species. Each model conceptualises thermal performance curves of various populations throughout the species' geographical range. From top to bottom, the curves represent populations from the warm edge to the cool edge. The width of the curve represents the fundamental (or ‘potential’) thermal niche of the population. Dashed lines suggest the critical thermal maximum (CTmax) in different populations. The green shadings represent the environmental temperatures experienced by the population. The grey shadings represent the thermal safety margin to cool extremes (left side) and warm extremes (right side). In panel a, the range of the fundamental niche and the upper thermal limit are the same in each population, resulting in decreasing thermal safety margins in warm‐edge populations. In panel b, the fundamental niche and the thermal limit of each population have adjusted to local environmental conditions, resulting in higher thermal limits in warm‐edge populations and similar thermal safety margins between populations.

Variation in thermal performance between individuals is widely recognised (Bennett et al. 2019; Bennett, Wernberg, Arackal Joy, et al. 2015; Araújo et al. 2013; Deutsch et al. 2008; Sunday et al. 2011), but broadscale evidence of how impacts of extreme warming (e.g., MHW) vary across the distribution of species remains low. Uncovering the degree to which structured differences exist between populations across a species range has important implications for predictions of sensitivity to climate extremes (Bennett et al. 2019). For instance, traditional species distribution modelling approaches often use the realised thermal distribution of a species as a proxy for the thermal conditions a species can tolerate, and analyse in turn how future warming may modify this distribution (Martínez et al. 2018; Stuart‐Smith et al. 2017; Stuart‐Smith et al. 2015). Such approaches typically assume that the thermal niche of individuals is conserved (i.e., remains the same, on average) across a species distribution, meaning that warm‐edge populations have less buffer between environmental temperatures and their upper thermal limits than populations living in cooler climes (niche conservatism model, Figure 1a).

However, physiological adjustment, such as local thermal adaptation (natural selection causing a modulation of the average phenotype within a population toward a local optimum) and phenotypic plasticity (the ability of producing different individual phenotypes in response to the environment), can contribute to differences in thermal limits between populations of a single species (Donelson et al. 2019; Fox et al. 2019; King et al. 2017). This forms the basis of the local adjustment model (Figure 1b), which conceptually contrasts with the niche conservatism model by assuming spatial variation in population‐level thermal niches. Eco‐physiological studies have long recognised differences in thermal performance between populations (Buckley et al. 2022; Wernberg, de Bettignies, et al. 2016; Howells et al. 2012; Somero 2010), potentially reducing the differences in sensitivity (i.e., thermal safety margins) between populations (e.g., Figure 1b). However, these studies tend to be conducted in controlled environments, and the extent to which results reflect responses in wild populations remains unresolved for many species (Dudney et al. 2025; Bates and Morley 2020).

Despite that the niche conservatism and local adjustment models suggest the binary response of species to a warming climate, the realised relationship between temperature and species performance is influenced by a range of environmental and ecological processes, such as nutrient limitation, competition and pollution (Fernández et al. 2020; Mabin et al. 2019; Filbee‐Dexter et al. 2016). The realised thermal performance likely falls between the adapted and non‐adapted patterns. In addition to the two primary theoretical approaches to predicting species' responses to warming (e.g., species distribution model and physiological experiments), realised impacts of MHWs have been widely observed in many foundation species (Smith et al. 2024; Wernberg et al. 2024; Wernberg, Bennett, et al. 2016). The realised dynamic responses of species from ecological surveys provide a more direct and accurate evidence of changes in species in response to the warming climate (Siegel and Dee 2025; Bennett, Wernberg, Arackal Joy, et al. 2015), even though it usually relies on the occurrence of spatially and historically limited climate extremes to capture significant responses.

Kelp are widespread foundation species, supporting coastal habitats in temperate regions (Smale 2020; Wernberg et al. 2019). In recent decades, substantial kelp forest losses have been caused by MHWs under climate change (Wernberg et al. 2024; Filbee‐Dexter and Wernberg 2018). Consolidating our understanding of kelp thermal performance and population‐level responses to MHWs is essential for improving predictions of climate extreme impacts, strengthening conservation efforts for the species and ecological functions they underpin. Here we used golden kelp (*Ecklonia radiata*) to empirically evaluate two theoretical frameworks—niche conservatism and local adjustment—by quantifying how realised thermal performance aligns with these conceptual models. Specifically, we analyse three decades of observed canopy cover data to detect the relationship between changes in kelp abundance and a range of MHW metrics. Additionally, we compare these patterns with experimental estimates of thermal performance across populations and ecological limits from species' geographic distributions. Each of the approaches offers a unique perspective on the species' population level sensitivity to MHW stress, illustrating the value of integrating physiological, ecological and biogeographic data to assess climate vulnerability. By comparing with long‐term reef monitoring program time‐series, we can assess the robustness of inferred thermal sensitivities and calibrate the relevance of experimental and distribution modelling predictions. Thus, this multi‐faceted approach better equips us to diagnose the causal impacts and responses to MHWs, which helps improve predictions of climate responses across species taxa and contribute more accurately toward recognising their vulnerability and risk from climate extremes.

## 2 Methods

### 2.1 Kelp Abundance

**FIGURE 2:**
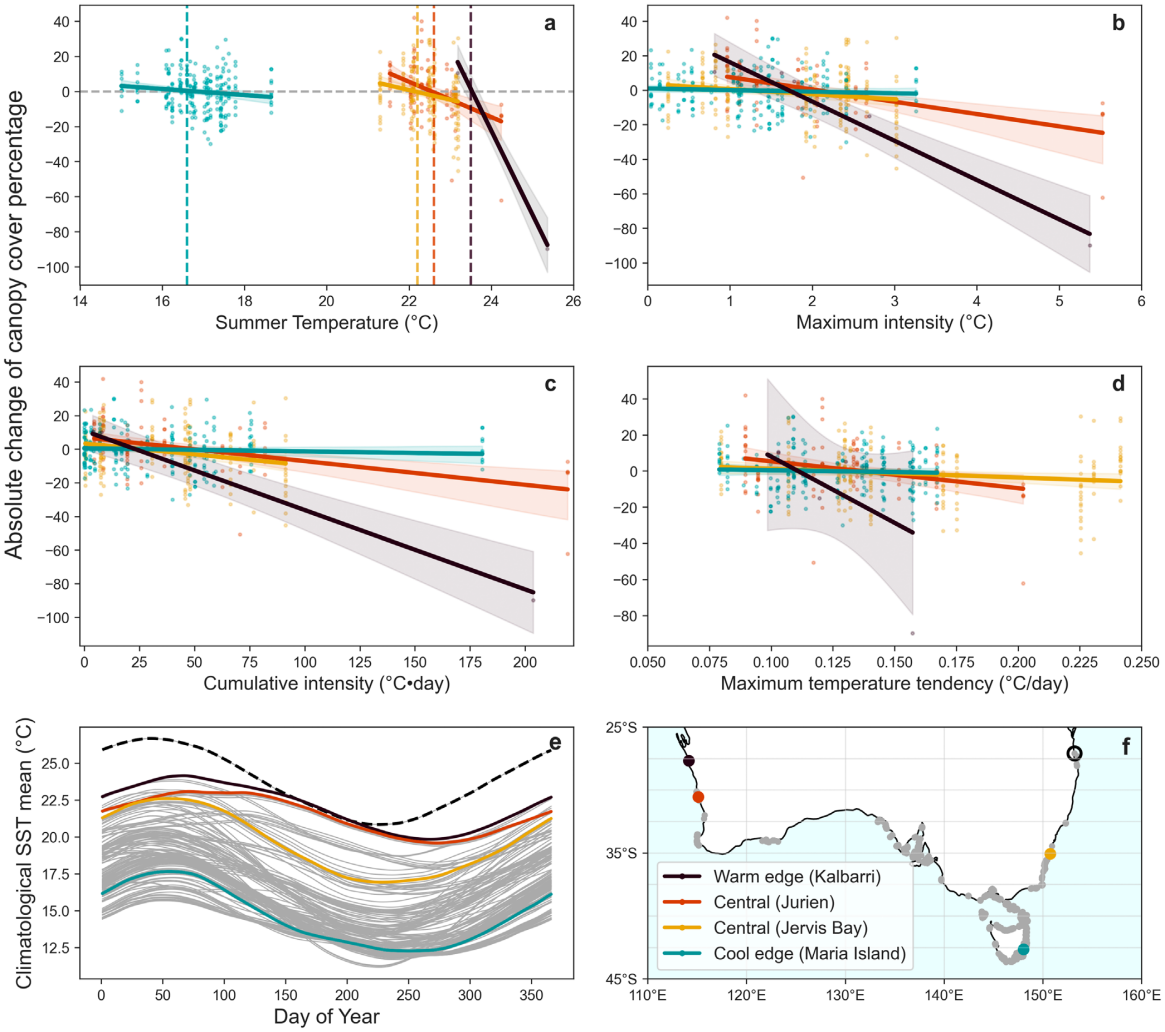
Observed kelp change in abundance in response to temperature and MHW metrics. Relationships between the annual change of surveyed kelp abundances and MHW metrics from the intervening summer (a: absolute temperature; b: maximum intensity; c: cumulative intensity; d: temperature tendency) during the past three decades at different populations. Raw observations are shown as scatters. Statistically significant solid fitting lines by GLMM represent local thermal response slopes of kelp canopy cover across different populations (*p* < 0.05). The shadings suggest 95% confidence intervals. Vertical dashed lines in panel a represent the mean of the warm‐season climatology in each location, which cross the zero canopy cover change for the cool‐edge and central locations. The historical observations from Kalbarri were reported in Wernberg, Bennett, et al. (2016). Panel (e) shows the seasonal climatological means of daily SSTs (1991–2021) for all study locations. Panel (f) shows the geographical distribution of *Ecklonia radiata* across southern Australia, as recorded in the Ocean Biodiversity Information System (grey points), together with the studied populations (coloured points). The black circle in (f) corresponds to the population with the highest temperature climatology (dashed black line in e), representing the location of the critical thermal maximum.

This study concentrates on southern Australia, home to the kelp forests that dominate Australia's Great Southern Reef (Bennett, Wernberg, Connell, et al. 2015). We use a high‐resolution long‐term seaweed abundance dataset from Australia's National Reef Monitoring Network (NRMN 2024). NRMN consolidates the reef biodiversity data, which have been collected using Underwater Visual Census techniques by a dedicated SCUBA dive team. Here we focused on the canopy cover abundance of golden kelp (*Ecklonia radiata*) over the past three decades during 1992–2023. We treated the kelp at different latitudinal locations as the populations at different range positions (Figure 2f), specifically Maria Island (42.5°S—cool‐edge), Jervis Bay (35.1°S—central), and Jurien (30.4°S—central), encompassing a total of 47 sites. At the warm edge, we incorporated data from three reefs at Kalbarri (27.7° S) collected from 2001 to 2012 (Wernberg, Bennett, et al. 2016). Details of survey methods and data treatment are provided in Supporting Information (see Table S1 for site‐level details). The relationship between MHWs and absolute annual kelp abundance is shown (Figure S1).

### 2.2 Warm Season Marine Heatwaves

We used sea surface temperature (SST) from High Resolution NOAA Optimum Interpolation 1/4 Degree Daily SST (OISST) Analysis, Version 2.1 (Huang et al. 2021) to conduct the MHW‐related analysis. Rather than focusing on Hobday et al. (2016) defined discrete MHW events above the seasonally varying 90th percentile threshold, we employed a set of temperature extremes metrics to characterise MHW in terms of magnitude, heating stress and abruptness (Supporting Information; Figure S2, Table S2; Gruber et al. 2021; Hobday et al. 2016), which offers a broader perspective on warming extremes relevant for kelp thermal stress. The baseline period for calculations of all MHW metrics utilised the OISST during the main kelp‐survey period (1991–2020). Given that climatological warm‐season temperature is widely utilised in forecasting coral bleaching events (Heron et al. 2016; Liu et al. 2014), and kelp tends to be resilient to milder thermal stress at the cooler environment and winter months (Martínez et al. 2018; Wernberg et al. 2010), here we focused on the warm‐season MHWs, specifically selecting the five warmest months (December—April). This 5‐month range captures the most intense and consistent warm conditions for all locations around southern Australia.

### 2.3 Kelp Abundance Change Response to MHWs

We analyse changes in *Ecklonia radiata* canopy cover across cool‐edge (Maria Island), central (Jervis Bay and Jurien) and warm‐edge populations (Kalbarri) over the past three decades to detect the relationship between MHWs and kelp abundance change. We compare the warm‐season MHWs with the changes in observed abundance before and after each summer during the whole survey period. To minimise temporal autocorrelation and avoid confounding by prior damage, we focus on the canopy cover change between consecutive surveys rather than raw canopy cover percentages. While this approach does not eliminate the effect of environmental stressors other than temperature, we expect thermal stress to be the leading order effect (Smale 2020; Wernberg, Bennett, et al. 2016). We note the surveys in Maria Island were undertaken in different months in each year, while Kalbarri, Jurien, and Jervis Bay were sampled consistently in December, October‐November and May‐June, respectively. To avoid potential seasonal difference, we concentrate on surveys in Mar‐May at Maria Island, the period with the highest survey frequency.

We applied general linear mixed‐effects models (GLMMs) to assess relationships between the canopy cover change and MHW metrics in three long‐term surveyed locations (Maria Island, Jervis Bay, Jurien). Site‐specific effects were treated as random variables to account for the hierarchical structure of local conditions, and temperature metrics were derived from OISST data averaged over a 0.5 × 0.5‐degree box covering all sites within each location. GLMMs were chosen for their robustness and interpretability in detecting spatial variation in thermal responses across locations. To assess temporal dependence, we calculated lag‐1 autocorrelation of residuals within sites among locations (Marshall et al. 2013). Correlations are small and on average slightly negative, with no evidence of consistent positive autocorrelation, indicating that residuals were generally independent across years. Since only location‐averaged data are available for Kalbarri, we used a generalised linear model to fit the relationship between kelp cover change and local MHW metrics. Furthermore, we tested alternative temperature metrics (e.g., warmest monthly temperature; Figure S4), which yielded similar results to those based on the mean daily warm‐season temperature.

### 2.4 Laboratory Experimented Thermal Physiological Response

**FIGURE 3:**
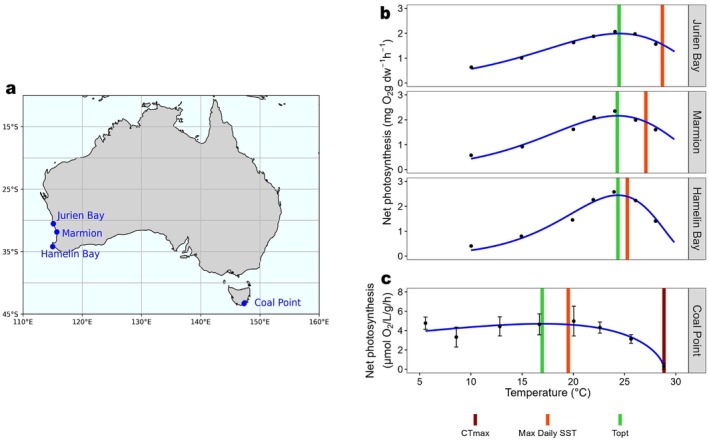
Thermal performance curves of kelp generated from laboratory experiments by Wernberg, de Bettignies, et al. (2016) in (b) and Britton et al. (2024) in (c), where the kelp were collected from different latitudinal locations (a). Thermal limits (critical thermal maximum—CTmax and optimal temperature—Topt) and the daily maximum of the SST observed by satellite are marked as vertical lines in different colours.

To compare the thermal performances of wild populations against those found in laboratory experiments, we synthesised data from two published studies that measured short‐term physiological thermal responses of *Ecklonia radiata* to temperature at locations across southern Australia (Britton et al. 2024; Wernberg, de Bettignies, et al. 2016). In these studies, kelp were collected by SCUBA divers at locations from Western Australia and Tasmania and transported to laboratories for experiments (Figure 3a). We extracted their recorded net photosynthesis data at locations from which thermal performance curves (TPCs) could be fitted for our focal locations. While the laboratory experiments (e.g., Wernberg, de Bettignies, et al. 2016) involved short‐term thermal exposure, our goal here is to compare the population‐level difference in thermal performance, specifically by focusing on the key parameters of TPC—critical thermal maximum (CTmax) as the temperature at which the fitted net photosynthesis curves cross zero (i.e., net photosynthesis became negative), and the optimum temperature (Topt) as the temperature yielding the maximum predicted net photosynthesis. TPCs were fitted following the approach of Britton et al. (2024), where the O'Neill et al. (1972) model provides the best fit for these datasets. Additionally, we compared these experiment‐derived thermal limits with the environmental temperature limits (i.e., maximum daily SST) using OISST from 1981 to 2022.

### 2.5 Thermal Range From Species Distribution

To obtain the thermal distribution of kelp, we used the records from the Ocean Biodiversity Information System (OBIS). We then combined OBIS records with OISST data (1991–2022) to reconstruct the thermal distribution of kelp (*Ecklonia radiata*) across southern Australia, highlighting four locations: Kalbarri, Jurien, Jervis Bay and Maria Island. Due to the difference in spatial resolution between the species' occurrence locations and SST data, we aggregated OBIS occurrences to match the ¼ degree resolution of OISST. If any species' occurrences were recorded within each ¼ degree grid cell, regardless of the number of records, the presence was noted as a single entity in each cell. The climatological mean SST at each presence was calculated to display the temperature range of species' distributions. We used the highest climatological mean SST across the species' presence records as a proxy for CTmax. While these realised upper limits are typically lower than the fundamental upper limits that are grounded in physiological tolerance studies, they are widely used to indicate climatic tolerance in the real world because they integrate the effects of chronic exposure and ecological constraints that ultimately determine species distributions in the wild (Pinsky et al. 2019; Stuart‐Smith et al. 2015; Sunday et al. 2012).

## 3 Results

### 3.1 Realised Impacts on Kelp Change

Over the past 30 years of survey observations around southern Australia, all kelp populations showed negative relationships between canopy cover change and metrics of summer temperatures and MHWs. These relationships are specifically characterised by increasing canopy cover in cool years and decreasing cover in warm years (Figure 2). Specifically, for all kelp populations, the modelled zero change occurred when summer temperatures were within the long‐term mean of warm‐season temperatures for the region, indicating that kelp loss is more likely triggered when summer temperatures exceed the mean climatology (Figure 2a).

Notably, populations differed significantly in their rates of decline in response to comparable temperature increase. We calculated the slopes of the regression lines that describe the relationship between the absolute percentage cover change and summer temperature (% °C^−1^) for each population, with associated 95% confidence intervals. Among the three long term programs monitoring reefs from central to cool edge populations, Jurien showed the steepest decline with increasing temperatures (−10.0% ± 2.5% per °C, *p* = 0.04), followed by a moderate response slope in the central populations (Jervis Bay: −5.6% ± 2.1% per °C, *p* = 0.02) and the lowest sensitivity in the cool‐edge population (Maria Island: −1.7% ± 1.8% per °C, *p* = 0.02). Moreover, data from a historically warm‐edge population at Kalbarri were consistent with this geographic pattern, exhibiting an even steeper decline with increasing temperature (−47.8%/°C ± 2.9% per °C, *p* < 0.001; Wernberg, Bennett, et al. 2016). The increasing rates that absolute canopy cover declined with temperature change from cool to warm‐edge populations corresponded with absolute summer temperatures (Figure 2a), but not for other MHW metrics (Figure 2b–d). Range position, therefore, plays an important role in determining the volatility of populations to absolute summer temperature. Here, volatility refers to the rate of change in canopy cover, representing the abruptness and magnitude of performance changes under thermal stress. Moreover, all four populations showed decreasing canopy cover change with increasing temperatures, consistently shifting from positive to negative change (Figure 2a). However, warm‐edge populations increased in abundance at temperatures above the threshold that typically triggers declines in cool‐edge populations.

While range position appeared to influence the sensitivity of kelp canopy cover to temperature change, the central (Jurien) population and historically warm‐edge population (Kalbarri) in Western Australia also experienced the highest MHW intensity (category IV; Hobday et al. 2018) on record during 2011 (Wernberg 2021; Wernberg, Bennett, et al. 2016), potentially confounding the effects of range position versus the magnitude of thermal anomaly. To test whether differences in responses between warm‐edge, central and cool‐edge populations were driven by their range positions or large MHW intensities, we reanalysed the relationships between absolute kelp cover change and summer temperatures for the two populations without the data corresponding to the 2011 MHW. Removing this largest anomaly resulted in the same pattern of decline with summer temperatures, with the populations closest to warm edges displaying steeper declines than the central and cool‐edge populations (Figure S5). We further tested the robustness of these slope estimates to different sample sizes among locations by randomly downsampling the larger datasets to match the smaller observation location. The resulting slope distributions from downsampled GLMM fits show consistent patterns with those from full datasets, whereby the kelp canopy cover decline slope in response to summer temperature increases toward warm edges (Figure S6). Together, these results confirm range position plays an important role in performance volatility of populations to absolute summertime temperature extremes, which implies the increasing vulnerability of populations as they approach the warm edge.

When examining the relationship with maximum and cumulative intensity, the warm‐edge population suffered the most substantial decline (Figure 2b,c), while the cool‐edge populations remained relatively stable. Warm‐edge and central populations experienced similarly high maximum and cumulative intensities, but displayed different rates of kelp cover change. This indicates that intensity metrics alone do not account for the different rates of kelp loss observed among populations. Temperature tendency measures the rate at which temperature extremes develop (Figure S2), which is particularly important in relation to species' resilience to abrupt temperature increases. The relationship between canopy cover change and temperature tendency (°C day^−1^) also follows the temperature gradient (Figure 2d), with the warmer edge populations displaying the steeper decline. This suggests that kelp, at their warmer edge, are more sensitive to the abruptness of temperature increases.

### 3.2 Laboratory Experiments on Physiological Performance

Laboratory thermal performances of kelp across southern Australia were constructed using physiological data of *Ecklonia radiata* from Britton et al. (2024) and Wernberg, de Bettignies, et al. (2016). Here, while the units are different from two experiments, we focused more on the critical thermal limits from thermal performance curves. Similar to field observations, thermal limits of kelp in laboratory‐controlled heating experiments follow the climatic conditions at their source location, with populations from lower‐latitude locations being more heat tolerant than those from higher‐latitude (poleward) locations (Figure 3). In contrast to the field observations, the CTmax predicted from laboratory experiments in the warm‐edge populations was > 40°C in west coast populations and 28.9°C in cool‐edge population on the east coast. For all locations, experimentally derived values of CTmax considerably exceed the environmental SST maximum, while the maximum daily SSTs were all higher than the Topt predicted from experiments. However, the 2011 MHW in Western Australia resulted in 100% kelp loss at Kalbarri (north of Jurien; Wernberg, Bennett, et al. 2016). The daily maxima temperature at Kalbarri as the realised upper threshold for *Ecklonia radiata* was 29.6°C, which is slightly above the maximum daily SST in Jurien (28.7°C). This realised temperature threshold for kelp collapse is far lower than the result derived from laboratory experiments, suggesting that the experimental CTmax overestimates the real upper thermal limits for species.

### 3.3 Thermal Distribution Model

In contrast with field observations and laboratory experiments, species distribution models assume a constant thermal tolerance across populations of a species (Smale and Wernberg 2013; Wernberg et al. 2013; Jordà et al. 2012). Climatological means for all four populations studied here follow a latitudinal gradient (Figure 2e,f). Thus, the thermal safety margin, calculated as the difference between the upper thermal limit and the experienced climatological SST, is narrower for warmer‐edge populations. This pattern aligns with the niche conservatism model. However, species distribution models do not account for the varying thermal performance thermal thresholds for kelp decline across different populations. Consequently, relying solely on a single proxy for thermal limits without identifying specific performance metrics overlooks the possibility of adaptation/acclimation among populations, a phenomenon that has recently been documented for *Ecklonia radiata* through modern genomics (Vranken et al. 2021).

## 4 Discussion

In this study we tested how a habitat‐forming kelp species responded to discrete temperature extremes (e.g., MHWs) in its natural environment across its geographical range. Unlike prior studies which typically assume a constant thermal niche or rely on laboratory experiments, we apply a multi‐faceted approach that integrates long‐term field observations to capture population‐level thermal sensitivity. By focusing on real‐world conditions, we evaluated the kelp changes associated with the complex and dynamic interactions that occur during MHWs. This research provides a more reliable understanding of kelp's thermal performance and a broadly applicable analytical framework for improving predictions of species’ vulnerability to future climate extremes.

**FIGURE 4:**
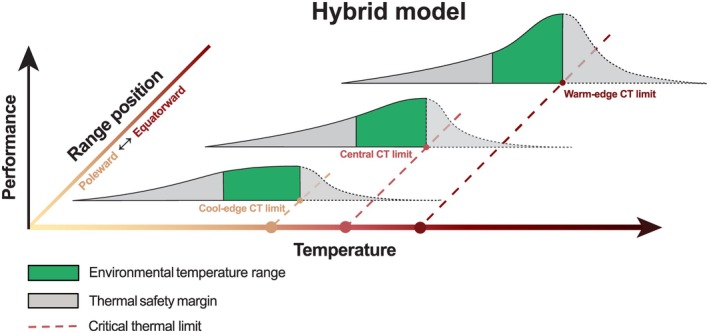
A hybrid model as the combination between local adjustment and niche conservatism models. The fundamental niche and the critical thermal limit (CT limit) for kelp decline of each population are adjusted to the local environmental conditions, but the performance within the environmental temperature experienced by warm‐edge populations is more volatile. The tails of the performance curves with dotted outlines reflect the uncertainties regarding future performances across populations with rising temperatures.

Our study of kelp performance in southern Australia, using three decades of reef monitoring data and daily SST, showed that change in kelp cover associated with MHWs was sensitive to the geographical range positions of populations. Warm‐edge populations were highly volatile to changes in temperature, exhibiting greater abruptness and magnitude in their thermal response, whereas cool‐edge populations exhibited lower rates of change to MHW conditions (Figure 2). This pattern suggests warm‐edge populations are more sensitive to extirpation than central and cool‐edge populations, consistent with the ‘niche conservatism’ model (Figure 1a). At the same time, each population displayed a negative relationship with temperature, with different thresholds for kelp loss corresponding to their respective summer climatological mean temperatures. This results in a gradient in temperature tipping points between kelp populations, reflecting characteristics more akin to the ‘local adjustment’ model (Figure 1b). Hence, our study bridges these theoretical concepts and the applications on kelp, pointing to a hybrid model which encapsulates both adapted and non‐adapted patterns positioned between niche conservatism and local adjustment. This hybrid model quantitatively illustrates the response of kelp at different populations to temperature extremes (Figure 4). Specifically, the warm‐edge population exhibited a more pronounced response to temperature changes than central and cool‐edge populations. At the same time, the shift from positive to negative canopy cover change supports the model prediction on population‐specific thermal thresholds. Particularly, the critical thermal limits varied between populations with thresholds for kelp decline increasing toward warmer edges, suggesting greater tolerance in warmer‐edge populations.

The hybrid model aligns with the latitudinal patterns in genetic diversity, where low‐genetic‐diversity kelp forests (populations at warm edges) are susceptible to a more abrupt collapse after similar MHWs, compared to cool‐edge populations with more diverse genotypes (Wernberg et al. 2018). Therefore, this different volatility of kelp response to MHWs among populations is likely a result of the underlying genetic diversity at different range positions (Bennett et al. 2019). Furthermore, local conditions may interact with MHWs to modulate their impacts on species, causing spatially varied biological responses to MHWs as well (Starko et al. 2024). Notably, the warm‐edge population increases in abundance above the threshold for decline in central and cool‐edge populations, underscoring the adapted behavior of warm‐edge populations. This is consistent with the intraspecific variability demonstrated in many physiological experiments of kelp (Strasser et al. 2022; Wernberg, de Bettignies, et al. 2016; Staehr and Wernberg 2009) and evidence of local adaptation in the range‐edge populations characterized by seascape genomics of *Ecklonia radiata* (Vranken et al. 2021).

The hybrid model provides a simplified framework for understanding population responses to MHW conditions in different range positions. We recognize that thermal performance can influence the range position of an individual, since species live where they physiologically can and end up where they adapt or acclimate. However, the focus of our study is on the observed population‐level thermal responses rather than evolutionary adaptation. We also cannot exclude the possibility of legacy effects of past MHWs, particularly where recovery may take multiple years. For example, kelp at Kalbarri has not recovered since the 2011 extreme Western Australian MHW (Wernberg 2021; Wernberg, Bennett, et al. 2016). However, across the other three locations, residual correlations of GLMMs were slightly negative, which indicates potential compensatory recovery rather than with persistent legacy effects. Together with the relatively stable variance in kelp cover at central and cool‐edge sites (Figure S1), this suggests that catastrophic legacy effects were unlikely during the study period, though this inference is constrained by the annual to biennial survey resolution. Future work with higher temporal resolution would be valuable to disentangle immediate responses from persistent legacy effects.

Our findings suggest that kelp partially adjust to local environment temperatures, but warm‐edge populations still have higher vulnerability to MHWs than those in the central and cool edges. Notably, the differences in the volatility across range positions have not been widely recognised in the marine realm. Previous work has largely focused on critical thermal limits, which overlook how organisms and populations vary in response around the thermal limits. The elevated volatility in warm‐edge populations highlights that critical thermal limits differ in consequences, with variation depending on the local temperature context. This in turn influences the chance of recovery or collapse of species. This is particularly informative for stakeholders in the context of conservation management and adaptation planning (Vranken et al. 2021; Wernberg et al. 2018), especially when temperatures exceed the threshold for triggering kelp decline. These findings also highlight a direction of future study investigating the potential for species populations to acclimate or adapt to warmer conditions and assessing whether such adjustments can keep pace with the accelerated rate of MHWs.

We found that the different volatility performance of kelp is more strongly determined by absolute summer temperature extremes than by anomaly magnitude alone. While variation in slope between populations could reflect differences in local temperature anomalies or other site‐specific stressors, removing the large anomaly associated with the extreme 2011 Western Australian MHW in Jurien and Kalbarri did not alter the relationship with absolute summer temperatures and warm‐edge populations still showed the largest volatility and the highest threshold for kelp decline (Figure S5). This reinforces that the declining pattern is statistically robust across all populations, despite the smaller dataset for Kalbarri (location‐averaged cover only; Wernberg, Bennett, et al. 2016), further supporting the same hybrid model. This finding aligns with the modelled relationship by Martínez et al. (2018), which identified summer temperatures as a critical predictor of kelp distribution in Australia. In relation to intensity metrics, warm‐edge populations exhibited a higher rate of kelp cover change, irrespective of anomaly magnitude. The pattern of differing slopes in kelp cover change is consistent with the pattern observed in the relationship to summer temperature, further confirming the robustness of the higher volatility of thermal performance observed in warm‐edge populations. We suggest that substantial kelp decline requires a maximum temperature anomaly exceeding 5°C (Figure 2b). However, we do not expect a consistent rate of kelp canopy cover change until its ultimate crash at the central and cool‐edge locations. It is also likely that cool‐edge populations will collapse at a lower threshold than warm‐edge populations (Bennett, Wernberg, Arackal Joy, et al. 2015), potentially due to the different magnitude of initial kelp cover or other environmental factors.

In addition to the primary analysis on the long‐term field observations, we employed a multi‐faceted framework by comparing the results with laboratory experiments and species distribution models to identify potential calibrations for examining the thermal impacts on kelp populations. In particular, the results of laboratory experiments exhibited a pattern consistent with the local adjustment model. Ocean temperatures had surpassed the species' Topt and performance decline should be imminent (Wernberg et al. 2024; Smale et al. 2019). However, such experiments may overestimate species' upper thermal limits, compared with the real‐world natural environment, which is subject to more complex compound stressors (Smith et al. 2025; Bass et al. 2021; Bennett, Wernberg, Harvey, et al. 2015). For example, laboratory experiments often keep nutrients constant. In reality, however, coastal warming often reflects stronger poleward boundary currents or warm‐core eddies with depleted nutrients (Holbrook et al. 2020; Holbrook et al. 2019). The East Australian Current is a nutrient‐depleted poleward flow, which can modulate kelp's thermal tolerance (Fernández et al. 2020; Johnson et al. 2011). Indirect effects of MHWs on kelp that can be neglected in experiments include herbivory from sea urchins and fishes (Bennett, Wernberg, Harvey, et al. 2015; Ling, Johnson, Frusher, and Ridgway 2009; Ling, Johnson, Ridgway, et al. 2009). Therefore, pairing the experimental insights with field observations is essential to capture the ecosystem‐level complexity and improve attribution of climate impacts (Dudney et al. 2025; Siegel and Dee 2025; Parmesan et al. 2013). In addition, we can obtain the thermal limits derived from species' geographic distributions to build species distribution models. However, this assumes a constant thermal niche within each species, but fails to consider the different performance between populations due to the local adjustment potentials (Klaassen et al. 2025; Valladares et al. 2014). Recent developments in modelling (e.g., demographic species distribution models) provide a promising way for integrating experimental, observational and distributional data to predict population‐level responses under climate change (Kotta et al. 2019; Ehrlén and Morris 2015). The importance of integrating experimental, observational and modelling approaches has been highlighted (Parmesan et al. 2013; Luo et al. 2011) which can help bridge the gap between multiple empirical approaches to assess species' vulnerability to climate extremes.

Reliable predictions of species responses to intensifying MHWs and warming extremes are crucial yet challenging for climate change ecology research. Our study provides empirical evidence of the varying vulnerability of populations across a species range to MHWs. By incorporating long‐term reef observations, we discovered that neither niche conservatism nor local adjustment alone adequately explains thermal responses of kelp populations to MHWs. Instead, our analyses converge on an empirically informed hybrid thermal performance model that blends elements of both and quantitatively characterises the population‐level responses. This work highlights the differences between realised impacts from thermal stress in natural populations and abstract predictions derived from thermal performance experiments or simple species distribution modelling approaches, which provides a basis for future efforts to calibrate the assessment of general principles of species' thermal performance. Therefore, we advocate this multi‐faceted analysis based on long‐term observations as a powerful and generalisable pathway for advancing predictions of thermal response to climate extremes (Reuman et al. 2025; Smith et al. 2022; Smale 2020). In the broader context of climate risk assessment, where sensitivity is a key dimension (Boyce et al. 2022; IPCC 2007), refining estimates of realised thermal performance under warming climate conditions through a comprehensive analytical framework will inform effective adaptation planning and climate risk management.

## Author Contributions

Jiaxin Shi, Scott Bennett, Jules B. Kajtar, and Neil J. Holbrook led the research design. Jiaxin Shi conducted analyses and manuscript development. Scott Bennett, Jules B. Kajtar and Neil J. Holbrook contributed the methodology and manuscript editing. Neville S. Barrett and Graham J. Edgar contributed to field data collection. Thomas Wernberg contributed to the Supporting Information from published sources. All authors discussed the results and contributed to the manuscript revision.

## Funding

This work was supported by Australian Research Council Centre of Excellence for Climate Extremes, CE170100023. Australian Research Council, DE200100900, DP200100201, FL240100015. Australia Research Council Centre of Excellence for the Weather of the 21st Century, CE230100012. Distinguished International Students Scholarship, 202206330007. Norwegian Research Council, GecoKelp project no. 335371. Ian Potter Foundation.

## Conflicts of Interest

The authors declare no conflicts of interest.

## Supporting information


**Appendix S1:** ele70307‐sup‐0001‐AppendixS1.pdf.

## Data Availability

All data and code that support the findings of this study are openly available in https://doi.org/10.5281/zenodo.17765732.
