# Supplementary material for: Warm Edge Kelp Populations Show Elevated Volatility to Marine Heatwaves
Source: Ecol Lett. 2026 Jan 23;29(1):e70307. doi: 10.1111/ele.70307 (PMC12828870; doi:10.1111/ele.70307)
Supplement: Supplementary file 1 — Appendix S1: ele70307‐sup‐0001‐AppendixS1.pdf. [file ELE-29-0-s001.pdf]

Supporting Information for

Warm edge kelp populations show elevated volatility to marine heatwaves

Jiaxin Shi [1,2], Scott Bennett [1], Jules B. Kajtar [3], Thomas Wernberg [4, 5], Neville S. Barrett [1], Graham J. Edgar [1], Neil J. Holbrook [1,2]

[1] Institute for Marine and Antarctic Studies, University of Tasmania, Hobart, Tasmania 7001, Australia
[2] Australian Research Council Centre of Excellence for the Weather of the 21st Century, University of Tasmania, Hobart, TAS, Australia
[3] National Oceanography Centre, Southampton, United Kingdom
[4] UWA Oceans Institute and School of Biological Sciences, University of Western Australia, Crawley 6009, WA, Australia.
[5] Institute of Marine Research, Flødevigen Research Station, His 4817, Norway

This PDF file includes:

Survey method details

Marine heatwave detection

Downsampling sensitivity test

Tables S1-2

Figures S1-6

Survey method details

This study focused on the coastal waters around southern Australia, and specifically on changes of kelp (*Ecklonia radiata*) cover observations over the past three decades. We compiled kelp abundance data in Maria Island, Jervis Bayt, and Jurien, primarily from the Australian Temperate Reef Collaboration (ATRC), accessed via Australia's National Reef Monitoring Network (NRMN). We additionally incorporated long-term monitoring data from Kalbarri as a supplementary dataset to extend spatial coverage (Wernberg et al. 2016a). All data were collected by trained SCUBA dive teams.

ATRC surveys

At ATRC sites, survey methods have been consistent across years and locations. Kelp cover is quantified using 0.25 m^2 point-intercept quadrats. Quadrats were placed every 10 m along a 50 m transect, with four transects per site, yielding 20 quadrats per survey. Surveys were typically conducted at 5 m and 10 m contour depths, though occasional surveys were conducted at depths ranging from 2 m to 12 m. Core sites were generally surveyed annually or biennially, starting from the same GPS points each time.

Kalbarri surveys

At Kalbarri, surveys were conducted at three reefs from 2001 to 2015 (Wernberg et al. 2016a). Methods slightly varied among years, including 6×1 m^2 quadrats (2005), 4×3.14 m^2 circular plots (2006–2009), and 10×0.25 m^2 quadrats per reef (post-2011). Values for 2001 and 2010 were estimated from in situ observations and interpolated from other pre-2011 measurements. Despite these differences, all methods provided comparable percentage estimates of kelp canopy cover. Since kelp in Kalbarri has not recovered since the 2011 Western Australian marine heatwave (MHW), we employed data only during 2001-2012. Here we employ the location-averaged data from Fig. 2C in Wernberg et al. (2016).

Data treatment and potential bias

We restricted our analyses to sites with at least three survey years, focusing on long-term monitored sites at different latitudes, which we treated as representing distinct populations across the species' range. While most sites were surveyed annually, some had occasional gaps. These gaps were not interpolated; analyses used only observed data. We acknowledge that uneven temporal coverage introduces potential biases, but by analysing population-level trends across regions rather than individual sites, we minimise the influence of local inconsistencies.

Table S1. Site-level survey effort for kelp populations included in this study. Total site-years refers to the number of unique site × year surveys used in the analysis.

Location	Population	Latitude	Longitude	Survey depth (m)	Number of sites	Survey period	Total site-years used in analysis	Source
Maria Island	Cool edge	42.5 ºS	148.1 ºE	5-10	10	1992-2023	217	ATRC
Jervis Bay	Central	35.1 ºS	150.7 ºE	2-10	24	1996-2023	194	ATRC
Jurien	Central	30.4 ºS	115.1 ºE	2-12	12	1999-2021	92	ATRC
Kalbarri	Warm edge	27.7 ºS	114.3 ºE	8-12	3	2001-2013	5*	Wernberg et al. (2016)

*To ensure consistency, we employed the location-averaged canopy cover time series presented in Wernberg et al. (2016).

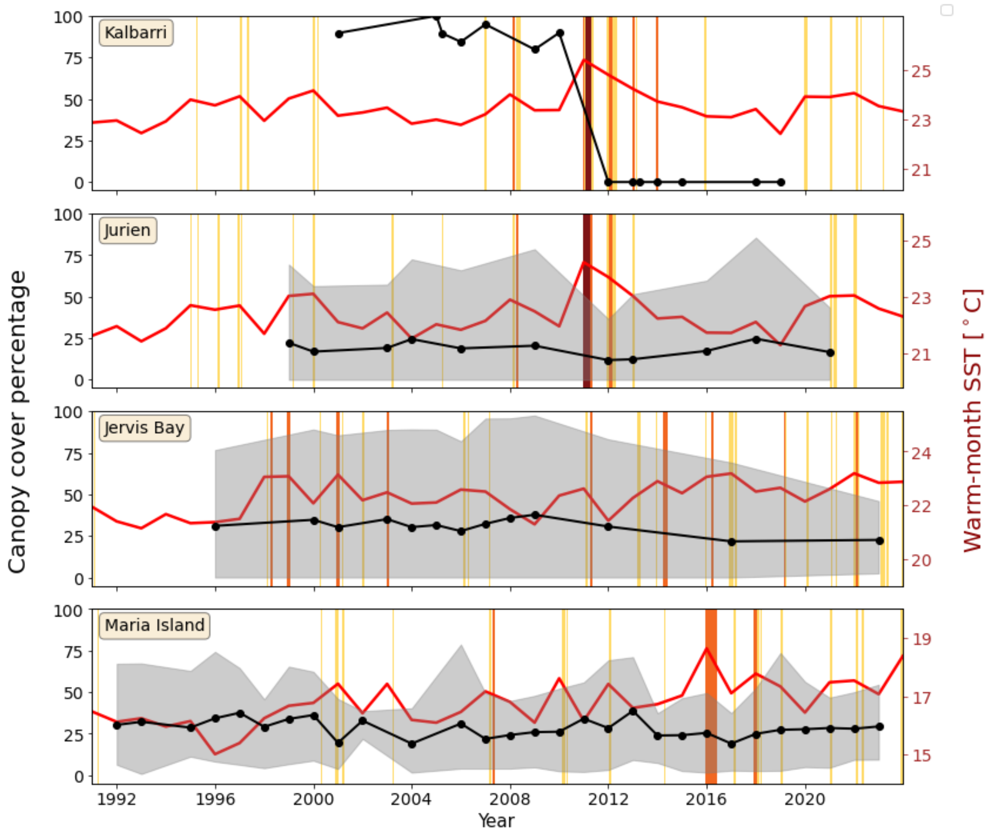

Figure S1. Timeseries of annual mean of warm-season sea surface temperature (SST; red) and canopy cover of *Ecklonia radiata* averaged at location level (black) in warm to cool locations (Wernberg 2021; Wernberg et al. 2016a), with indication of different categories of warm-season MHW events (Hobday et al. 2018). The grey shadings are the canopy cover spread of different sites within each location.

Marine heatwave detection

In the first instance, MHWs were detected following the Hobday et al. (2016) definition – MHW events are identified when SST exceed the seasonally varying 90th percentile threshold climatology (based on the fixed 30-year baseline period from 1983-2012) for at least five consecutive days.

For the calculation of MHW intensity metrics here, however, we adopted a modified approach. Instead of the seasonally varying climatology used by Hobday et al. (2016), we defined the baseline threshold as the maximum of the daily seasonal climatology, which represents a more ecologically critical level. This choice reflects evidence that kelp loss is more strongly associated with absolute temperature thresholds than with statistically anomalies (Filbee-Dexter et al. 2020; Cavanaugh et al. 2019). Specifically, the daily seasonal SST climatology was first computed, and its maximum was used as the reference threshold for quantifying maximum and cumulative intensities, with respect to which the maximum and accumulated intensities were determined (Figure S2; Table S2). This approach emphasises ecologically critical thermal stress levels rather than statistical exceedances.

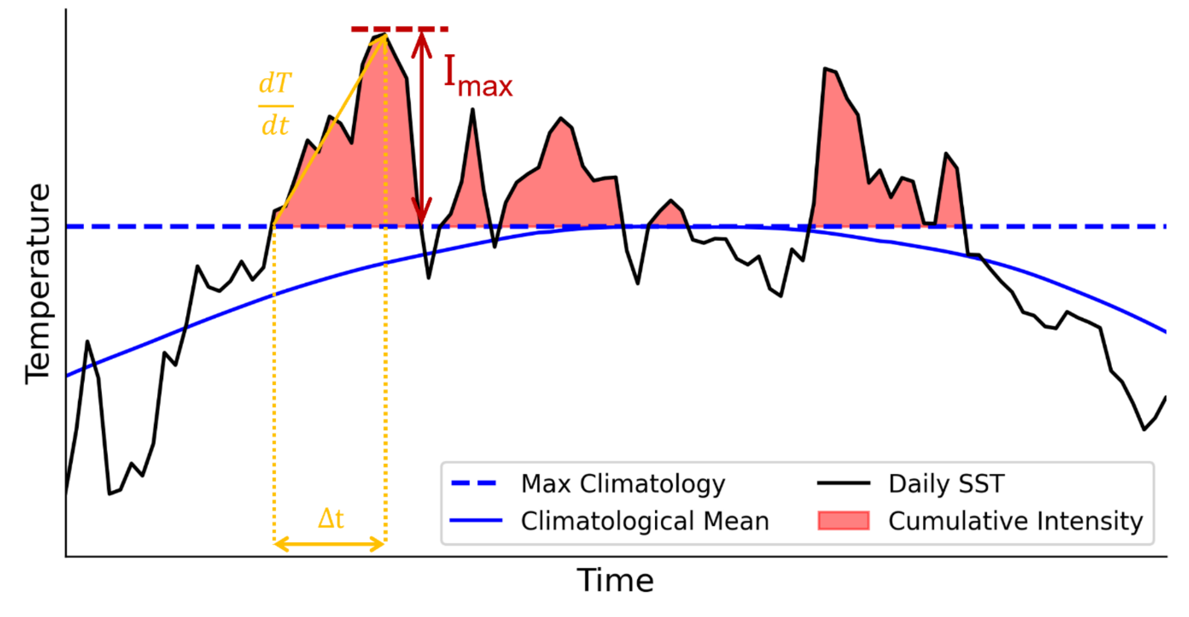

Figure S2. Schematic of the climatological maxima for identifying maximum intensity (I_{max}), cumulative intensity, and temperature tendency (Gruber et al. 2021; Hobday et al. 2016).

Table S2. MHW metrics (Gruber et al. 2021; Hobday et al. 2016). N is the total number of days in the warm season; T_i is the daily SST during the warm season; $Clim_{max}$ is the temperature climatological maximum.

Variable	Description	Equation
Absolute Temperature (°C)	Warm-season annually averaged temperature	$\frac{1}{N}\sum_{i=1}^{N} T_i$
Maximum intensity (°C)	Maximum positive anomaly in daily temperatures of the warm season relative to the climatological maximum in each year (Figure S2).	$\max\,(T_i - Clim_{max})$
Cumulative intensity (°C·day)	Summed positive daily temperature anomalies in the warm season relative to the maximum climatology in each year (Figure S2).	$\sum_{i=1}^{N}(T_i - Clim_{max})$
Maximum temperature tendency (°C·day^{-1})	Maximum rate of every 10-day mean temperature increase in warm season	$\max\,(\frac{T_{i+10} - T_i}{10})$

Temperature data validation

Although OISST is widely used for ocean surface temperature analysis in oceanography, we validated it against subsurface temperatures around kelp survey depths, using the Bluelink Ocean Reanalysis (BRAN2020; Chamberlain et al. 2021). The results showed strong correlations between the subsurface temperatures and SSTs across the study locations (Figure S3).

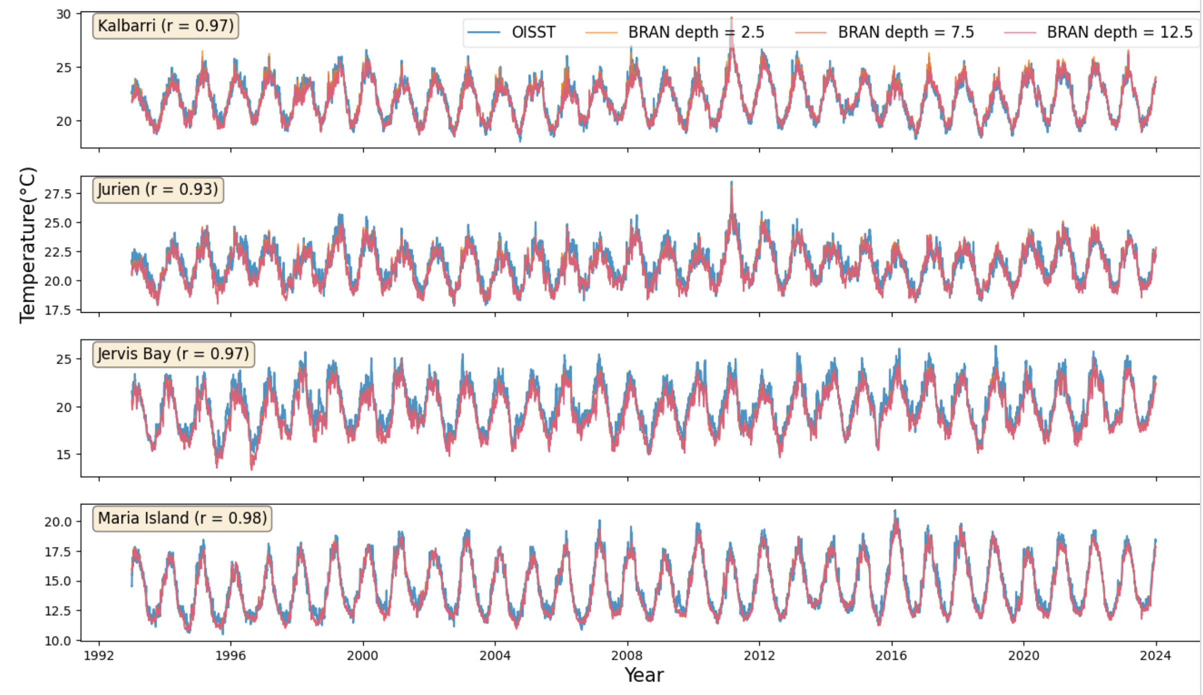

Figure S3. Comparison of timeseries of the U.S. National Oceanic and Atmospheric Administration Optimum Interpolation SST and the Bluelink Ocean Reanalysis ocean temperatures at depths (Chamberlain et al. 2021) at each studied location. *r* refers to the average of correlation coefficients between SST and ocean temperatures at different depth.

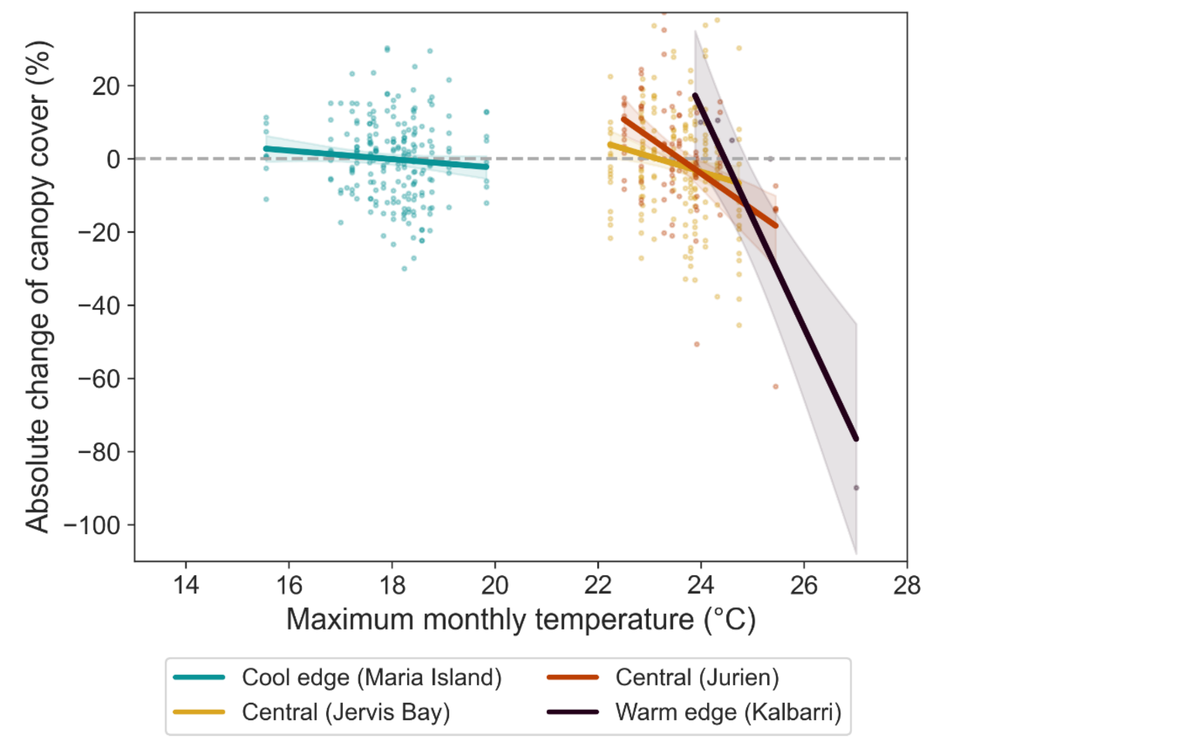

Figure S4. Relationships between the change in kelp abundance and warmest monthly temperatures.

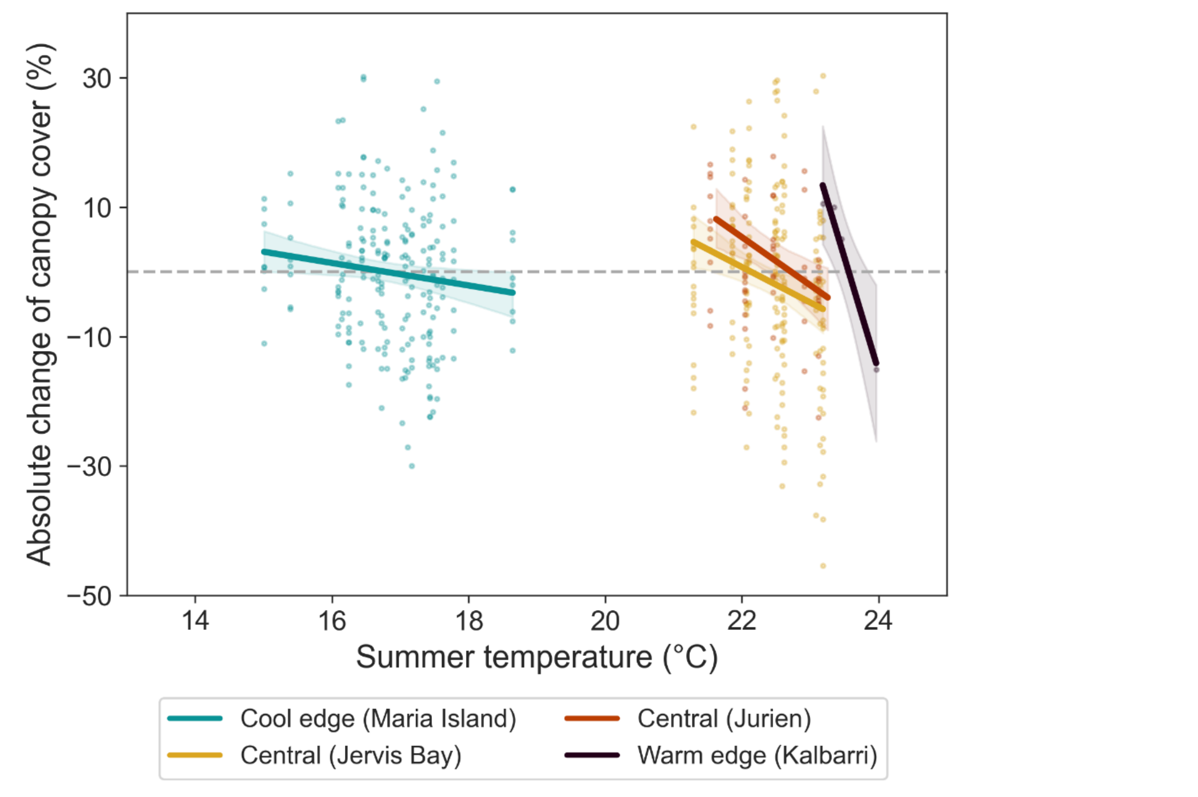

Figure S5. Relationship between kelp abundance change and warm season daily mean temperatures without data in 2011 for Jurien and Kalbarri.

Downsampling sensitivity test

To assess the sensitivity of temperature effects on kelp cover change to differences in sample size among locations (Table S1), the data points for Maria Island and Jervis Bay were randomly downsampled to 92 to match the number of observations at Jurien. This downsampling was repeated 10 times, and general linear mixed-effects models were refitted for each replicate. The distribution of slopes across replicates was used to evaluate the robustness of the estimated summer temperature effects (Figure S6).

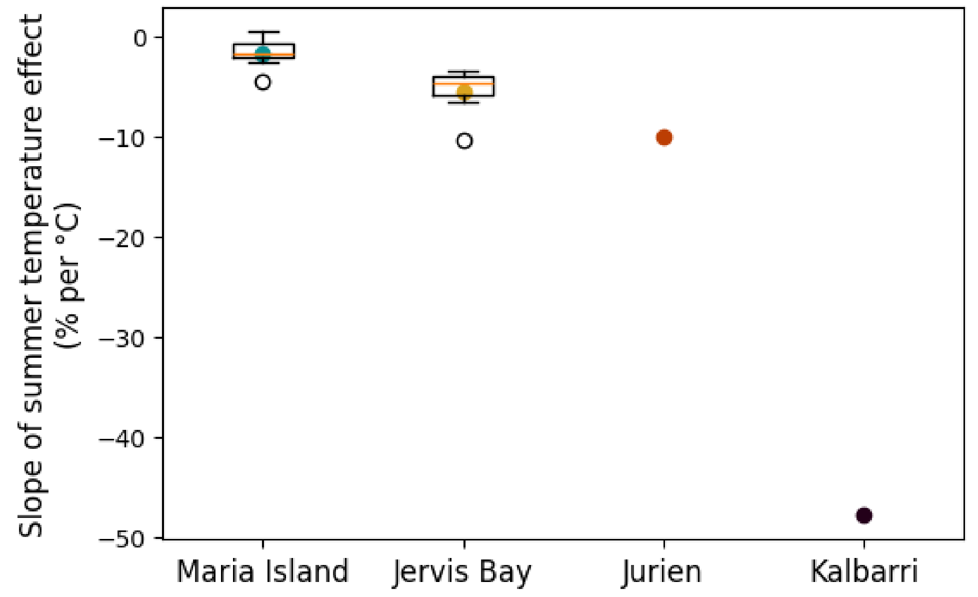

Figure S6. Slopes of kelp canopy cover decline in response to increasing summer temperatures are shown in colored dots. Slopes were estimated using general linear mixed-effects models for Maria Island, Jervis Bay, and Jurien, and a generalised linear model for Kalbarri. For Maria Island and Jervis Bay, the datasets were downsampled to match the number of Jurien data points using 10 bootstrap replicates. The resulting slopes from these replicates are shown as boxplots, with the mean slope across replicates indicated by an orange horizontal line.

References

Cavanaugh, K.C., Reed, D.C., Bell, T.W., Castorani, M.C.N. & Beas-Luna, R. (2019). Spatial Variability in the Resistance and Resilience of Giant Kelp in Southern and Baja California to a Multiyear Heatwave. *Frontiers in Marine Science*, 6.

Chamberlain, M. A., Oke, P. R., Fiedler, R. A. S., Beggs, H. M., Brassington, G. B., & Divakaran, P. (2021). Next generation of Bluelink ocean reanalysis with multiscale data assimilation: BRAN2020. *Earth Syst. Sci. Data*, 13(12), 5663-5688.

Filbee-Dexter, K., Wernberg, T., Grace, S.P., Thormar, J., Fredriksen, S., Narvaez, C.N. et al. (2020). Marine heatwaves and the collapse of marginal North Atlantic kelp forests. *Sci Rep*, 10, 13388.

Gruber, N., Boyd, P. W., Frolicher, T. L., & Vogt, M. (2021). Biogeochemical extremes and compound events in the ocean. *Nature*, 600(7889), 395-407.

Hobday, A. J., Alexander, L. V., Perkins, S. E., Smale, D. A., Straub, S. C., Oliver, E. C. J., et al. (2016). A hierarchical approach to defining marine heatwaves. *Progress in Oceanography*, 141, 227-238.

Hobday, A. J., Oliver, E. C. J., Gupta, A. S., Benthuysen, J. A., Burrows, M. T., Donat, M. G., et al. (2018). Categorizing and Naming MARINE HEATWAVES. *Oceanography*, 31(2), 162-173.

Wernberg, T., Bennett, S., Babcock, R. C., Bettignies, T. d., Cure, K., Depczynski, M., Dufois, F., Fromont, J., Fulton, C. J., Hovey, R. K., Harvey, E. S., Holmes, T. H., Kendrick, G. A., Radford, B., Santana-Garcon, J., Saunders, B. J., Smale, D. A., Thomsen, M. S., Tuckett, C. A., . . . Wilson, S. (2016). Climate-driven regime shift of a temperate marine ecosystem. *Science*, 353(6295), 169-172.
